# The ER-alpha mutation Y537S confers Tamoxifen-resistance via enhanced mitochondrial metabolism, glycolysis and Rho-GDI/PTEN signaling: Implicating TIGAR in somatic resistance to endocrine therapy

**DOI:** 10.18632/aging.101690

**Published:** 2018-12-20

**Authors:** Marco Fiorillo, Rosa Sanchez-Alvarez, Federica Sotgia, Michael P. Lisanti

**Affiliations:** 1 Translational Medicine, School of Environment and Life Sciences, Biomedical Research Centre (BRC), University of Salford, Greater Manchester M5 4WT, United Kingdom; 2 The Department of Pharmacy, Health and Nutritional Sciences, The University of Calabria, Cosenza, Italy; 3 University of Manchester, The Paterson Institute, Manchester M1 39PL, United Kingdom

**Keywords:** estrogen receptor, Y537S mutation, TIGAR, hormone therapy, mitochondria, drug resistance

## Abstract

Naturally-occurring somatic mutations in the estrogen receptor gene (ESR1) have been previously implicated in the clinical development of resistance to hormonal therapies, such as Tamoxifen. For example, the somatic mutation Y537S has been specifically associated with acquired endocrine resistance. Briefly, we recombinantly-transduced MCF7 cells with a lentiviral vector encoding ESR1 (Y537S). As a first step, we confirmed that MCF7-Y537S cells are indeed functionally resistant to Tamoxifen, as compared with vector alone controls. Importantly, further phenotypic characterization of Y537S cells revealed that they show increased resistance to Tamoxifen-induced apoptosis, allowing them to form mammospheres with higher efficiency, in the presence of Tamoxifen. Similarly, Y537S cells had elevated basal levels of ALDH activity, a marker of “stemness”, which was also Tamoxifen-resistant. Metabolic flux analysis of Y537S cells revealed a hyper-metabolic phenotype, with significantly increased mitochondrial respiration and high ATP production, as well as enhanced aerobic glycolysis. Finally, to understand which molecular signaling pathways that may be hyper-activated in Y537S cells, we performed unbiased label-free proteomics analysis. Our results indicate that TIGAR over-expression and the Rho-GDI/PTEN signaling pathway appear to be selectively activated by the Y537S mutation. Remarkably, this profile is nearly identical in MCF7-TAMR cells; these cells were independently-generated *in vitro*, suggesting a highly conserved mechanism underlying Tamoxifen-resistance. Importantly, we show that the Y537S mutation is specifically associated with the over-expression of a number of protein markers of poor clinical outcome (COL6A3, ERBB2, STAT3, AFP, TFF1, CDK4 and CD44). In summary, we have uncovered a novel metabolic mechanism leading to endocrine resistance, which may have important clinical implications for improving patient outcomes.

## Introduction

In human breast cancer patients, the hormone estrogen and its main receptor (ESR1), are key drivers of tumor initiation, cancer progression and ultimately metastasis [1-4]. As a consequence, targeted therapies, such as Tamoxifen, were first developed to inhibit estrogen receptor signaling, in ER(+) breast cancer cells [5]. Historically, Tamoxifen represents one of the earliest forms of targeted therapy and was first clinically trialed in the 1970’s, at the Christie Hospital in Manchester, UK [6-8].

Unfortunately, however, endocrine therapy ultimately fails in a significant number of patients on long-term anti-estrogen therapy, due to the acquired emergence of drug-resistance. The resulting treatment failure often has dire consequences for the patient, with the emergence of tumor recurrence and distant metastasis, resulting in poor clinical outcomes.

If we are going to successfully prevent or reverse treatment failure, we need to know the underlying mechanism(s) by which ER(+) tumor cells can successfully escape the effects of hormonal therapy. Until recently, the prominent role of somatic mutations in the estrogen receptor, in conferring resistance to endocrine therapy, has remained largely unappreciated [4,9].

These somatic mutations can significantly change the conformation and functional activity of the estrogen receptor, effectively locking it a constitutively-activated state [10]. Two of the most common mutations, namely Y537S and D538G, both allow the estrogen receptor to bind coactivators, in the absence of the estrogen ligand, resulting in a constitutively-active receptor [11-13].

Here, we mechanistically studied the phenotypic effects of the Y537S mutation on MCF7 cells in culture. For this purpose, we created a genetic model in MCF7 cells by stably over-expressing the ESR1 cDNA carrying the Y537S mutation. Importantly, we show that the Y537S mutation confers a hyper-active phenotype, due to the metabolic re-programming of mitochondrial function and the glycolytic pathway, resulting in increased ATP production and resistance to apoptosis, effectively protecting CSCs from the anti-mitochondrial effects of Tamoxifen.

Interestingly, it is well-known that Tamoxifen also functions as an inhibitor of mitochondrial complex I activity [14,15]. Therefore, it is perhaps not surprising that Tamoxifen resistance could be achieved, by the ability of the Y537S mutation to effectively augment mitochondrial “power”.

In support of this hypothesis, high levels of key mitochondrial markers, including complex I proteins, are specifically-associated with Tamoxifen-resistance in human breast cancer patients [16].

## RESULTS

### Generating a genetic model of Tamoxifen-resistance: MCF7-Y537S cells

Somatic mutations of the human estrogen receptor alpha (ESR1) have been directly implicated in the pathogenesis of hormonal therapy resistance in human breast cancer patients [1,9]. However, the exact mechanism(s) by which these ESR1 mutations induce Tamoxifen-resistance remains largely unknown. To begin to dissect how these mutations phenotypically confer drug resistance, we chose to construct an *in vitro* genetic model, using MCF7 cells, an ER(+) breast cancer cell line.

Briefly, MCF7 cells were transduced with a lentiviral vector carrying the Y537S mutation of ESR1 and positive “pools” of cells were selected, using a puromycin resistance cassette. Four other isogenic MCF7 cells lines were also generated in parallel, which served as negative controls for these experiments: ESR1 (WT and Y537N), ErbB2 and empty-vector (EV).

**Figure 1:**
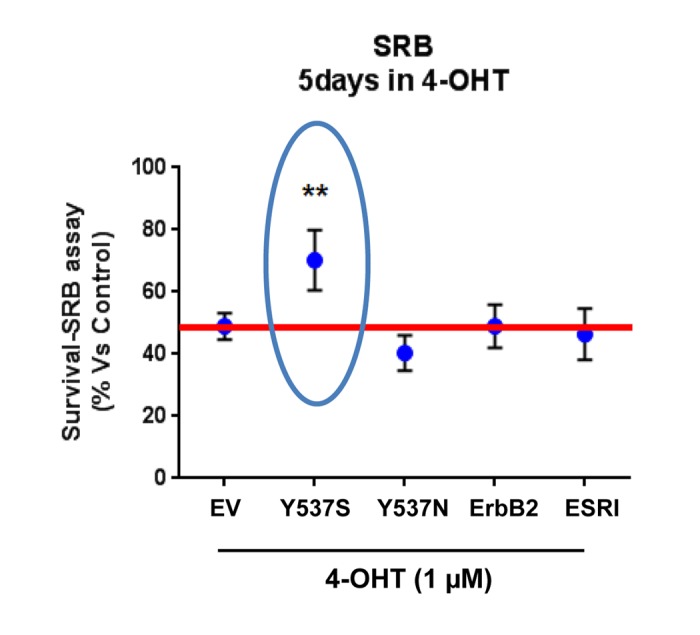
**Lentiviral transduction with the ESR1 (Y537S) mutation is sufficient to stably confer Tamoxifen-resistance in MCF7 cell monolayers: Effects on cell viability.** Briefly, MCF7 cells were stably-transduced with either ESR1 (WT, Y537S, or Y537N) or ErbB2 (HER2), to genetically create a clinically relevant model of hormone therapy resistance. Vector alone control MCF7 cells were generated in parallel (empty vector; EV; p-EV-105-puroR), as a negative control. Importantly, note that MCF7-Y537S cells clearly show resistance to 4-OHT (1 µM). The SRB assay was performed as a measure of cell viability and the experiment was carried out for 5 days. In contrast, 4-OHT has significant inhibitory effects on the viability of the other MCF7 cell lines. ** p<0.005.

To directly determine the validity of our model system, MCF7-Y537S cells were cultured for 5 days in the presence of Tamoxifen (1 µM) to assess its affect on cell viability. Importantly, Figure 1 shows that only MCF7-Y537S cells manifest a Tamoxifen-resistance phenotype, while all the other MCF7 cell lines tested remained completely Tamoxifen-sensitive.

These findings provide the necessary evidence for the use of MCF7-Y537S cells as a valid genetic model of Tamoxifen-resistance. Since the Y537N mutation did not drive Tamoxifen resistance in this context, other micro-environmental factors may be needed to observe this phenotype.

### Y537S drives resistance to Tamoxifen-induced apoptosis, enhancing mammosphere formation

An additional mechanism by which the Y537S mutation may contribute to Tamoxifen-resistance is its potential effect(s) on “stemness” and/or apoptosis.

**Figure 2:**
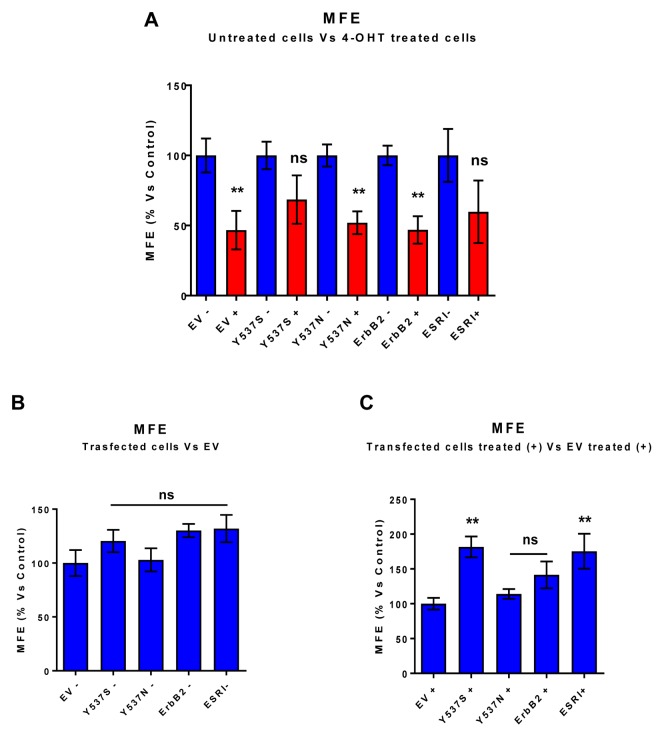
**MCF7-Y537S cells are resistant to the inhibitory effects of Tamoxifen on mammosphere formation: Quantitation.** Mammosphere formation assays were carried out for 5 days, in 6 well-plates, under low-attachment conditions. All the transfected MCF7 cell lines were grown as mammospheres. Note that 72h of pre-treatment with 4-OHT (1 µM) inhibits mammosphere formation efficiency (MFE), in all transfected cell lines, with the exception of MCF7-Y537S and MCF7-ESRI (WT) cells. In contrast, no changes in mammosphere formation were observed in the absence of 4-OHT (1 µM) pre-treatment. ** p< 0.005; ns = not significant evaluated by Student’s t test. (**Panel A**) Treated (RED) vs. Untreated (BLUE); (**Panel B**) Untreated; (**Panel C**) Treated with 4-OHT. EV, empty vector control; +, plus Tamoxilen; -, no Tamoxilen.

**Figure 3:**
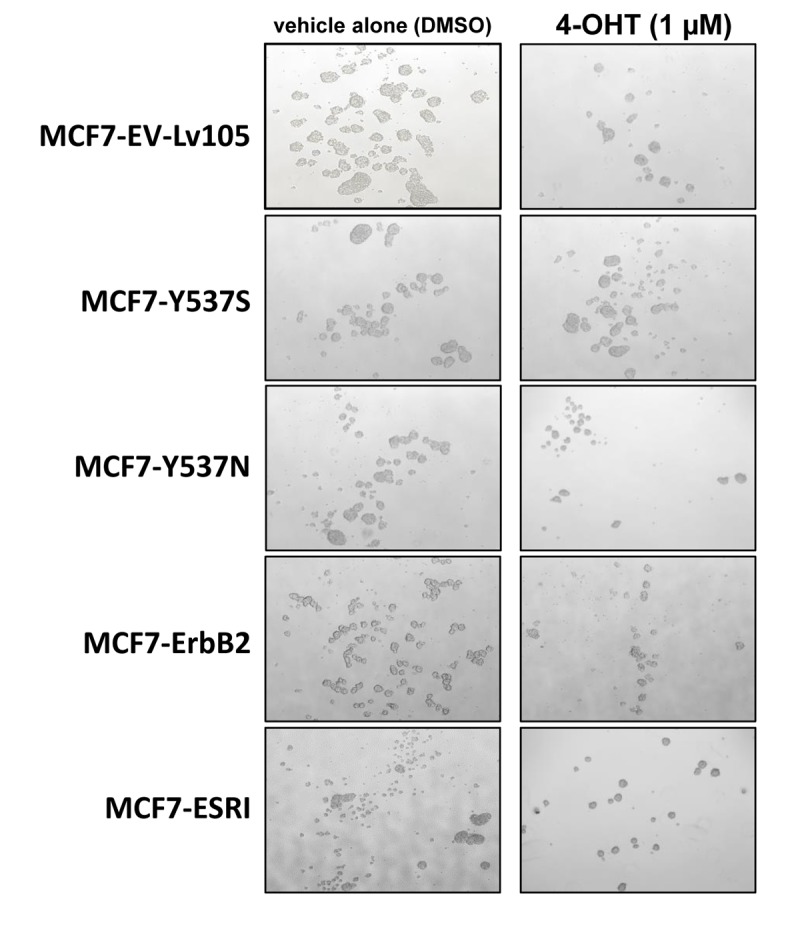
**MCF7-Y537S cells are resistant to the inhibitory effects of Tamoxifen during mammosphere formation: Representative images.** Note that overall 4-OHT (1 µM) treatement reduces mammosphere formation; however, MCF7-Y537S cells remain largely unaffected. Representative images are shown. The MCF7-Y537S cells show an obvious resistance to 4-OHT. The images were obtained with an Olympus microscope (4X objective, bright field).

To test this hypothesis, we first assessed potential effects on CSC propagation, using the mammosphere assay. In the absence of Tamoxifen, the Y537S mutation had no effect on mammosphere formation. However, in the presence of Tamoxifen, the Y537S mutation significantly promoted mammosphere formation, by nearly 2-fold. However, similar effects were also observed with the wild-type ESR1. Quantitation of these results is presented in Figure 2 and representative images are shown in Figure 3.

**Figure 4:**
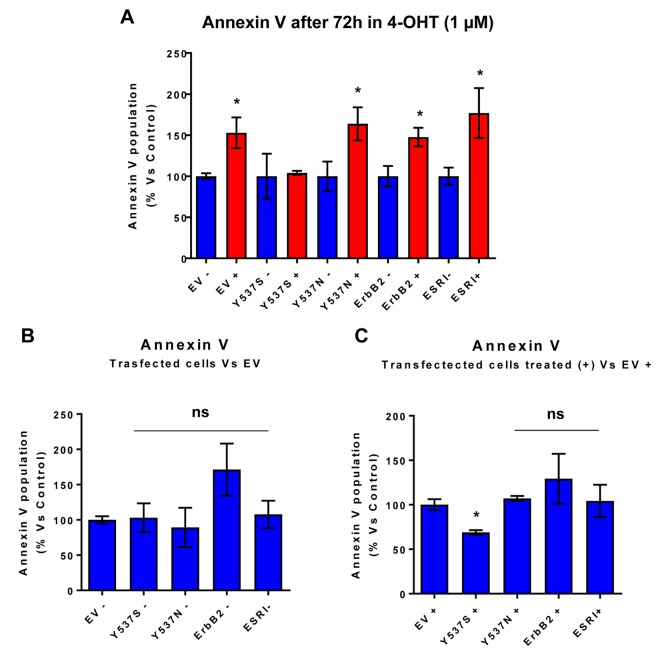
**MCF7-Y537S cells are resistant to the pro-apoptotic effects of 4-OHT**. Briefly, the transduced MCF7 cell lines were all plated in 6-well plates. On the next day, the cells were treated with 4-OHT (1 µM) for 72 hours. MCF7-EV cells were processed in parallel, as a negative control. Bar-graphs were used to summarize the overall results. Note that annexin V levels were increased in all transfected cell lines; however, MCF7-Y537S cells were specifically resistant to the pro-apoptotic effects of 4-OHT. * p<0.05. In contrast, no changes in Annexin V levels were observed in all transfected cells, in the absence of 4-OHT (1 µM); ns = not significant. (**Panel A**) Treated (RED) vs. Untreated (BLUE); (**Panel B**) Untreated; (**Panel C**) Treated with 4-OHT.

One mechanism by which the Y537S mutation may promote mammosphere formation in the presence of Tamoxifen is by conferring resistance to apoptosis. In direct support of this hypothesis, Figure 4 highlights that the Y537S mutation significantly reduces annexin-V staining in the presence of Tamoxifen, as revealed by FACS analysis, consistent with apoptosis resistance.

**Figure 5:**
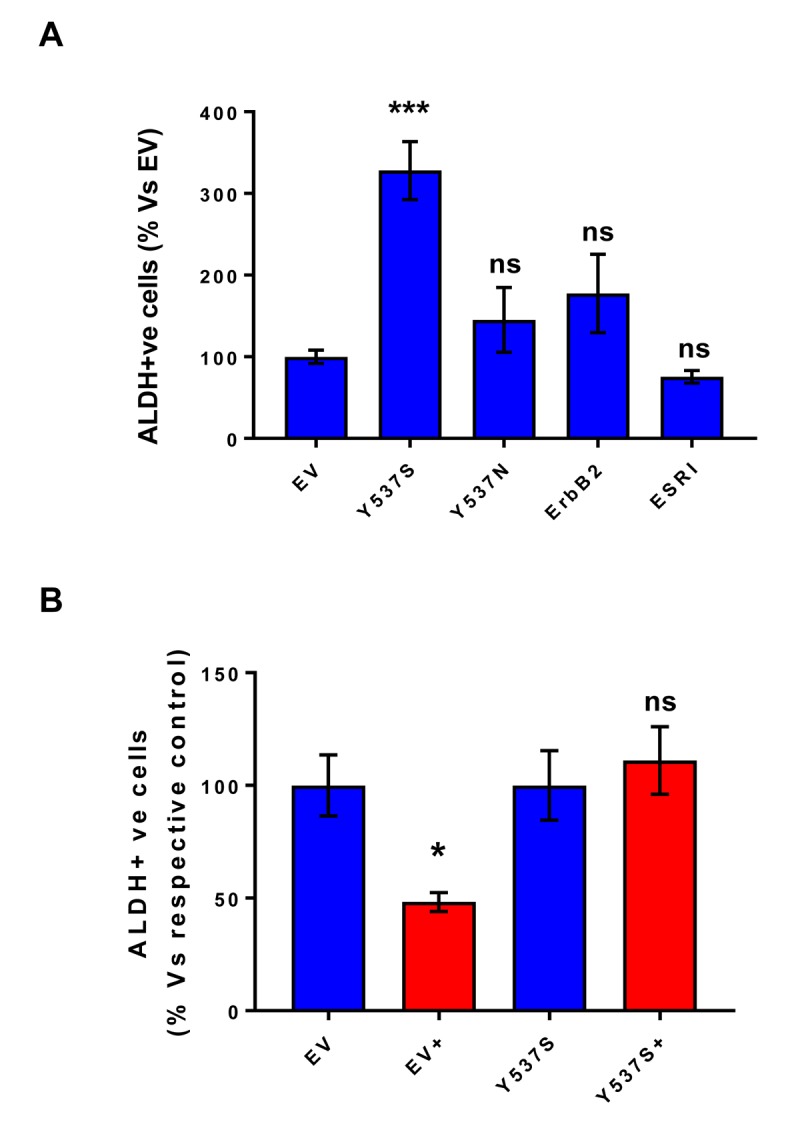
**MCF7-Y537S cells show increased ALDH activity, which is resistant to Tamoxifen treatment.** All the transfected MCF7 cell lines were first grown as mammospheres and then used to prepare a single-cell suspension, which was subjected to “stemness” assays with ALDEFLOUR, to measure ALDH activity. Importantly, only Y537S cells show significant increases (>3-fold) in ALDH activity. Also, note that treatment with 4-OHT (1 µM) inhibits ALDH activity in empty vector (EV) control cells, but not in MCF7-Y537S cells. * p< 0.05; *** p< 0.0005; ns = not significant, as evaluated by Student’s t test. (**Panel A**) ALDH activity; (**Panel B**) ALDH activity, with and without treatment with 4-OHT. This observation is consistent with the idea that increased stemness helps to confer tamoxifen-resistance.

As a second independent marker of “stemness”, we next assessed the levels of ALDH activity, by using ALDEFLUOR as a fluorescent probe, during FACS analysis. Briefly, all the transfected MCF7 cell lines were first grown as mammospheres and then used to prepare a single-cell suspension. Importantly, only Y537S cells showed significant increases (>3-fold) in ALDH activity (Figure 5A). Also, note that treatment with 4-OHT (1 µM) inhibits ALDH activity in empty vector (EV) control cells, but not in MCF7-Y537S cells (Figure 5B). This observation is consistent with the idea that increased “stemness”, driven by the Y537S mutation, helps to confer tamoxifen-resistance.

### Y537S confers a hyper-metabolic phenotype, with increased mitochondrial function and ATP production, elevated mitochondrial biogenesis and enhanced glycolysis.

We next hypothesized that one phenotypic mechanism by which the Y537S mutation may confer Tamoxifen-resistance is via the process of metabolic re-programming.

**Figure 6:**
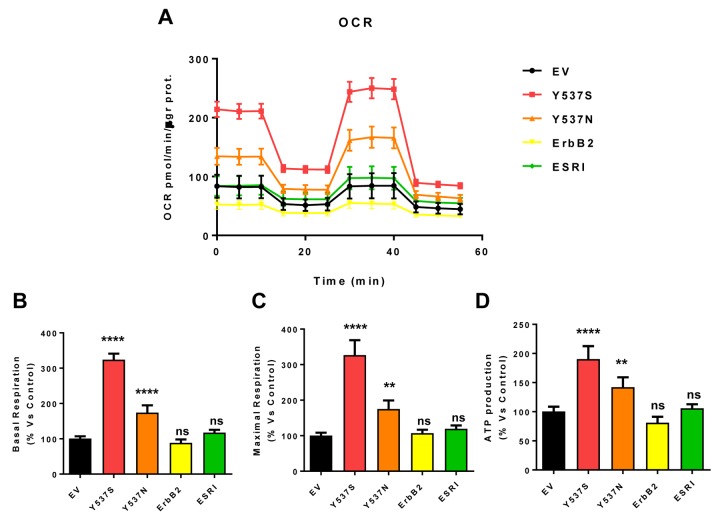
**MCF7-Y537S cells show a significant increase in mitochondrial oxygen consumption rate (OCR) and ATP production.** The Seahorse XFe96 metabolic-flux analyzer was employed to determine mitochondrial function in all of the MCF7 cell transfectants, after 48 hours of pre-treatment with 4-OHT (1 µM). (**Panel A**) A representative line graph of 3 independent experiments is shown (+/- SEM). **(Panels B, C** and **D**) Note that respiration (basal and maximal), as well as ATP levels, were significantly increased in MCF7-Y537S and MCF7-Y537N cells. However, MCF7-Y537S cells showed that largest increases. ** p < 0.001; **** p < 0.00001; ns = not significant.

**Figure 7:**
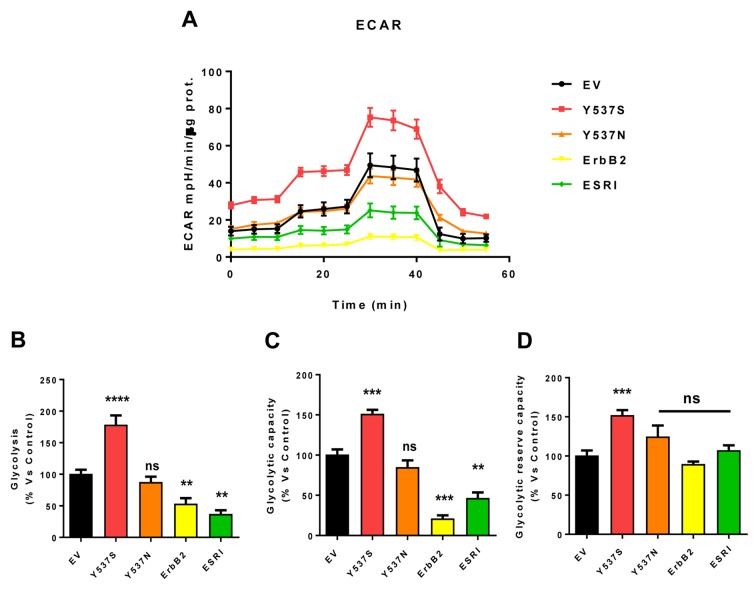
**MCF7-Y537S cells show a significant increase in extracellular acidification rate (ECAR) and glycolysis levels.** The Seahorse XFe96 metabolic-flux analyzer was employed to determine the metabolic function of all transfected cells after 48 hours of treatment with 4-OHT (1 µM). **(Panel A**) A representative line graph of 3 independent experiments is shown (+/- SEM). (**Panel B**) Glycolysis was significantly increased only in MCF7-Y537S cells and reduced in MCF7-ErbB2 and MCF7-ESRI cells. (**Panel C**) Glycolytic capacity was significantly increased only in MCF7-Y537S and reduced in MCF7-ErbB2 and MCF7-ESRI cells. (**Panel D**) Glycolytic reserve capacity was significantly increased only in MCF7-Y537S cells. ** p < 0.001; *** p 0.0001; **** p < 0.00001; ns = not significant.

As a consequence, we subjected Y537S cells to metabolic phenotyping with the Seahorse XFe96 metabolic flux analyzer. Figures 6 and 7 show the results of these studies. Importantly, we observed that the Y537S mutation significantly increases the mitochondrial oxygen consumption rate (OCR) and ATP production, by >3-fold and ~2-fold, respectively. Similarly, the Y537S mutation also substantially elevated glycolysis and the glycolytic reserve capacity, by nearly 2-fold. Therefore, MCF7-Y537S cells are hyper-metabolic, with enhanced mitochondrial and glycolytic function.

**Figure 8:**
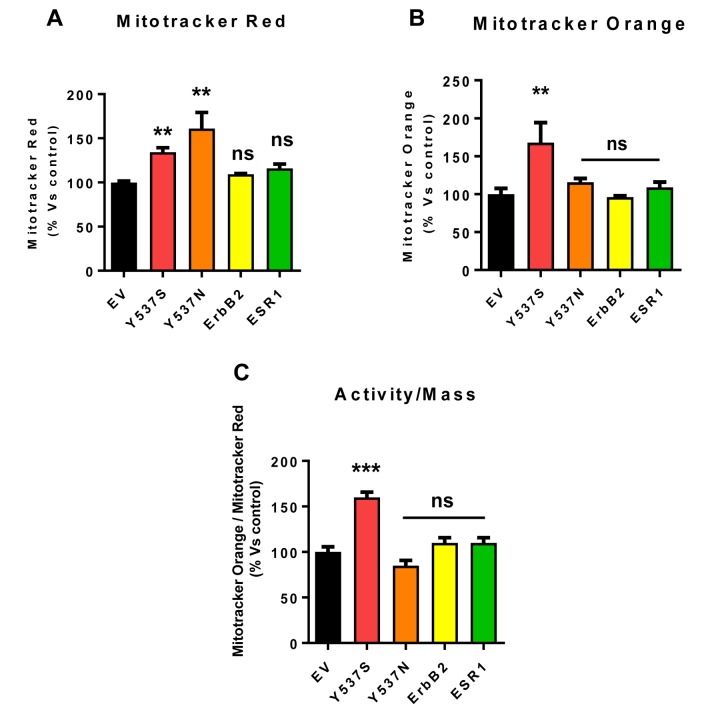
**Mitochondrial biogenesis and membrane potential are increased in MCF7-Y537S cells, in the presence of 4-OHT**. To determine the possible effects of the Y537S mutation on mitochondrial biogenesis and membrane potential, MCF7-Y537S cells were subjected to flow-cytometry, using MitoTracker probes. FACS analysis was carried out on MCF7 transfected cells after pre-treatment with 4-OHT. (**Panels A** and **B**) Note that MCF7-Y537S and MCF7-Y537SN cells show a significant increase in mitochondrial mass (MitoTracker Deep-Red), but an increased mitochondrial membrane potential (MitoTracker Orange) was observed only in MCF7-Y537S cells in growth media with 4-OHT (1 µM). (**Panel C**) Finally, the ratio (activity/mass) of mitochondrial membrane potential (MitoTracker Orange) and mitochondrial mass (MitoTracker Deep-Red) was increased only in MCF7-Y537S cells, in growth media containing 4-OHT. ** p < 0.001; *** p 0.0001; ns = not significant.

This elevated mitochondrial function may be due to increased mitochondrial biogenesis. Figure 8 illustrates that the Y537S mutation increases both mitochondrial mass and mitochondrial membrane potential, as observed by flow-cytometry with MitoTracker probes. This approach allowed us to calculate the activity/mass ratio, which was also increased in MCF7-Y537S cells.

Remarkably, previous studies with TAMR cells showed similar metabolic re-programming, with increased OCR and ATP production, as well as elevated mitochondrial biogenesis [17]. TAMR cells are an MCF7-based model of Tamoxifen-resistance, generated via the long-term culture of MCF7 cells in the presence of increasing concentrations of Tamoxifen [17,18].

### Proteomics analysis reveals that the Y537S mutation up-regulates key metabolic targets and hyper-activates Rho-GDI/PTEN signalling in MCF7 cells.

To further validate the hyper-metabolic phenotype that we observed via Seahorse analysis, we next subjected MCF7-Y537S cells to unbiased label-free proteomics analysis. For example, MCF7-Y537S cells were compared to MCF7-ESR1(WT) and empty-vector alone control cells, all in the presence of Tamoxifen.

Relative to ESR1(WT), the ESR1(Y537S) mutant showed dramatic increases in 33 mitochondrial proteins, consistent with increased mitochondrial oxygen consumption (OXPHOS) and elevated mitochondrial biogenesis (Supplementary Table S1). Several key components of Complex I were also up-regulated (NDUFB10, NDUFV1, NDUFA5). In addition, the ESR1(Y537S) mutant showed significant elevations in glycolytic and PPP enzymes (Supplementary Table S2), including TIGAR, which has been previously shown to be sufficient to confer Tamoxifen-resistance. In this context, TIGAR expression was infinitely up-regulated by the ESR1(Y537S) mutation. Interestingly, the ESR1(Y537S) mutant was specifically associated with high levels of seven markers of poor clinical outcome (COL6A3, ERBB2, STAT3, AFP, TFF1, CDK4, CD44) (Supplementary Table S3). Thus, a single point mutation in the estrogen receptor can drive extensive metabolic re-programming.

**Figure 9:**
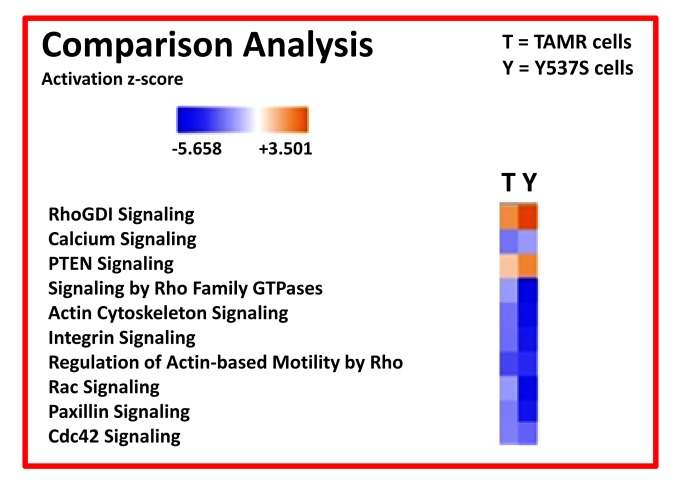
**Ingenuity Pathway Analysis (IPA) of proteomics data sets obtained from two distinct Tamoxifen-resistant breast cancer cell lines (TAMR vs. MCF7-Y537S cells).** The canonical signaling pathways predicted to be altered are shown. Briefly, both 4-OHT resistant cell lines (TAMR vs. MCF7-Y537S) were compared to each other, as well as with MCF7 control cells, all grown as monolayers. HeatMaps of the top 10 regulated canonical pathways are shown. A positive z-score (Orange) points towards the activation of a signalling pathway, while a negative z-score (Blue) indicates the inhibition of a pathway (p < 0.05 and cutoff z-score ± 2).

We also compared the profile of MCF7-Y537S cells with MCF7-TAMR cells, another more established Tamoxifen-resistant cell line. TAMR cells were originally derived by chronically culturing MCF7 cells in low levels of Tamoxifen, and then increasing its concentration in a step-wise fashion over time. Remarkably, these extensive comparisons revealed that a Rho-GDI/PTEN signaling pathway appears to be hyper-activated in both of these Tamoxifen-resistant cell lines (Figure 9; see also Figures S1 to S4).

### Clinical relevance of metabolic marker proteins induced by the Y537S mutation: Predicting the response to endocrine therapy.

To further validate the clinical relevance of our findings, we next assessed whether the “metabolic targets” that we identified in MCF7-Y537S cells were also transcriptionally upregulated in human breast cancer cells in vivo. For this purpose, we employed a published clinical data set of N=28 breast cancer patients in which their tumor samples were subjected to laser-capture micro-dissection (25), to physically separate epithelial cancer cells from their adjacent tumor stroma. Supplementary Table S4 presents a summary of these findings. Overall, many of the “metabolic targets” that we identified were also transcriptionally elevated in human breast cancer cells *in vivo*.

Next, we determined if the metabolic proteins up-regulated by the Y537S mutation in MCF7 cells have any clinical prognostic value, for predicting the onset of tamoxifen-resistance in breast cancer patients. For this purpose, we examined their mRNA expression levels in a cohort of ER(+)-patients. This population consisted of 152 patients, with the most common sub-type of breast cancer (Luminal A), with local lymph-node (LN) metastasis at diagnosis, specifically undergoing endocrine therapy (mostly Tamoxifen), without any form of chemotherapy.

In this setting, Tamoxifen-resistance manifests itself clinically, as either i) tumor recurrence or ii) distant metastasis. As such, Kaplan-Meier (K-M) curves were constructed using recurrence-free survival (RFS) or distant-metastasis free survival (DMFS), over a period of 10 to 15 years of follow-up. Hazard-ratios (HR) and p-values (log-rank test) were calculated and are as shown in Supplementary Tables S5-S8.

More specifically, Supplementary Tables S5 and S7 highlight the mitochondrial mRNA transcripts associated with tumor recurrence and distant metastasis, respectively. Similarly, Supplementary Tables S6 and S8 show the glycolytic and PPP enzyme mRNA transcripts associated with poor clinical outcomes.

Note that the mRNA levels of key metabolic proteins induced by the Y537S mutation positively predict treatment failure during endocrine therapy, highlighting their clinical relevance. These findings provide a direct functional link between the Y537S mutation, metabolic re-programming and the clinical response to endocrine therapy.

## DISCUSSION

### Understanding how the Y537S mutation promotes Tamoxifen-resistance

Somatic mutations in the estrogen receptor gene are specifically associated with the onset and development of hormone therapy resistance in human breast cancer patients [1,3,5]. In particular, the Y537S mutation drives endocrine resistance by maintaining the estrogen receptor in the constitutively activated state, resulting in an aggressive clinical phenotype, leading to tumor recurrence and distant metastasis [19,20].

In this report, we generated a new cellular model of endocrine therapy resistance, by modeling the gain-of-function effects afforded by the acquisition of the Y537S mutation. For this purpose, using a lentiviral vector, we inserted the cDNA encoding the mutated ESR1 (Y537S) gene into MCF7 human breast cancer cells. As negative controls, a series of other isogenic MCF7 cell lines, harboring the wild-type estrogen receptor and the empty-vector (EV), were also generated. Importantly, we first functionally validated that expression of the ESR1 (Y537S) mutant was indeed sufficient to experimentally confer Tamoxifen-resistance, relative to other control cell lines, tested side-by-side. Remarkably, the Y537S mutant conferred drug-resistance to Tamoxifen-induced cell apoptosis, allowing the efficient formation of 3D tumor spheroids, even in the presence of Tamoxifen.

We also tested the hypothesis that the Y537S mutation confers an abnormal metabolic phenotype, reflecting a form of gene-induced metabolic re-programming. In particular, by using the Seahorse XFe96 metabolic flux analyser, we determined the effects of the Y537S mutation on the i) oxygen consumption rate (OCR) and ii) the extracellular acidification rate (ECAR), as well as ATP production. Interestingly, the Y537S mutation resulted in a hyper-metabolic state, accompanied by elevated rates of mitochondrial respiration, enhanced ATP levels and increased glycolysis. Consistent with these findings, the Y537S mutation also increased mitochondrial mass and membrane potential, likely reflecting an increase in mitochondrial biogenesis.

Unbiased proteomics analysis was carried out to identify the key metabolic targets that were increased by the Y537S mutation. Ultimately, over 30 nuclear-encoded mitochondrial proteins were found to be over-expressed, as well as greater than 9 enzymes linked to glycolysis and the pentose-phosphate pathway.

Interestingly, the Y537S mutation was also linked to the over-expression of a number of protein markers of poor clinical outcome (TIGAR, COL6A3, ERBB2, STAT3, AFP, TFF1, CDK4, CD44). Ingenuity Pathway Analysis (IPA) independently demonstrated that the proteomic profile of MCF7-Y537S cells is very similar to MCF7-TAMR cells, another Tamoxifen-resistant cell line created by chronic exposure to Tamoxifen [17]. Both cell lines show the hyper-activation of a Rho-GDI/PTEN signaling pathway. These novel mechanism(s) driving Tamoxifen-resistance clearly have important implications for significantly improving clinical outcomes for breast cancer patients.

### Linking the Y537S mutation to TIGAR

TIGAR (TP53-inducible glycolysis and apoptosis regulator) was originally discovered as a p53-regulated gene [21,22]. However, TIGAR shows striking protein sequence similarity to the glycolytic enzyme that degrades fructose-2,6-bisphosphate, especially within its bisphosphate domain [23,24]. Therefore, TIGAR likely functions as an inhibitor of glycolysis, but also stimulates the up-regulation of the pentose-phosphate pathway (PPP) and can confer protection against apoptosis [22,23].

Interestingly, we have previously shown that the expression of TIGAR is indeed sufficient to confer Tamoxifen-resistance [25]. Therefore, since the expression of TIGAR is infinitely up-regulated by expression of the Y537S mutation, this could also help explain the mechanism by which Y537S confers Tamoxifen-resistance.

### Association of the Y537S mutation with COL6A3

Collagen VI is an extracellular matrix protein that has been previously associated with tumor progression and distant metastasis [26]. Since the expression of COL6A3 is infinitely up-regulated by expression of the Y537S mutation, this could also explain how Y537S confers Tamoxifen-resistance. Tumor-specific isoforms of COL6A3 have been reported [27].

### Metabolic markers induced by the Y537S mutation determine the response to endocrine therapy

Finally, we determined if the metabolic proteins up-regulated by the Y537S mutation in MCF7 cells have any clinical prognostic value, for predicting the onset of tamoxifen-resistance in breast cancer patients (i.e., tumor recurrence and/or distant metastasis). Importantly, our results indicate that the mRNA levels of key metabolic proteins induced by the Y537S mutation positively predict treatment failure during endocrine therapy, highlighting their clinical relevance. These findings provide a direct functional link between the Y537S mutation, metabolic re-programming and the clinical response to endocrine therapy.

### Potential metabolic therapies for targeting Tamoxifen-resistance

As our current results demonstrate that MCF7 cells harboring the Y537S mutation show characteristic features of metabolic re-programming and increased “stemness”, this suggests that patients harboring the Y537S mutation could ultimately benefit from treatment with metabolic therapeutics, specifically targeting i) mitochondrial metabolism, ii) glycolysis and iii) NAD(+) recycling (enumerated in Table 1). In previous studies, we have shown that several FDA-approved antibiotics (Doxycycline, Tigecycline, Azithromycin, Pyrvinium pamoate, Atovaquone and Bedaquiline) and natural products (Actinonin, CAPE, Berberine, Brutieridin and Melitidin) can all be used to effectively target mitochondria in ER(+) breast cancer stem cells (CSCs) [28–34]. Moreover, glycolysis inhibitors, such as Vitamin C (Ascorbic acid), 2-deoxy-glucose (2-DG), Silibinin, and Stiripentol, were also effective at targeting ER(+) breast CSCs [33,34]. In addition, our clinical pilot study (Phase II) has already shown that Doxycycline pre-treatment is effective in ER(+) breast cancer patients, for significantly reducing CSCs (by up to 66.67%), with a response rate of nearly 90% [35]. We anticipate that MCF7 cells recombinantly over-expressing the ESR1-Y537S mutation will provide a new fruitful isogenic model for the metabolic screening of these and other candidate drugs, for combating Tamoxifen-resistance (Table 1).

**Table 1:** Potential metabolic therapies for targeting Tamoxifen-resistance.

**1. Mitochondrial Inhibitors**	**References**
• Doxycycline	[28–30]
• Tigecycline	[28,30]
• Azithromycin	[28,30]
• Pyrvinium pamoate	[28,30]
• Atovaquone	[30,31]
• Bedaquiline	[30,32]
• Actinonin	[30,33]
• CAPE (Caffeic Acid Phenyl Ester)	[30,33]
• Berberine	[30,34]
• Brutieridin & Melitidin (isolated from Bergamot)	[30,37]
	
**2. Glycolytic Inhibitors**	
• Vitamin C (Ascorbic acid)	[30,33,34]
• 2-Deoxy-Glucose (2-DG)	[30,33,34]
• Silibinin	[30,33]
• Stiripentol	[30,33,34]
	
**3. NAD(+) Inhibitors**	
• FK-866	[30,33]

## CONCLUSIONS

**Figure 10:**
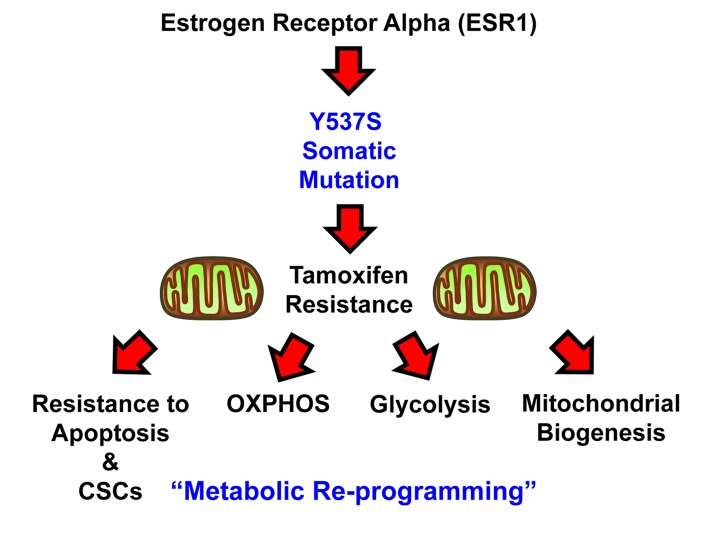
**Schematic diagram summarizing the role of the ESR1-Y537S mutation in driving Tamoxifen-resistance.** Note that the Y537S mutation induces metabolic reprogramming, with the hyper-activation of both mitochondrial and glycolytic energetic pathways.

In conclusion, here we have generated a genetic model of Tamoxifen-resistance in MCF7 cells, by over-expressing a somatic mutation of the estrogen receptor, namely ESR1-Y537S. Our results directly show that the Y537S mutation confers Tamoxifen-resistance by driving re-programming towards a hyper-active metabolic phenotype, characterized by high levels of OXPHOS and mitochondrial biogenesis, as well as elevated levels of glycolysis (Figure 10). This contention is supported by genetic evidence, quantitative metabolic flux analysis, flow-cytometry, unbiased proteomics and computer-assisted bioinformatics analysis. These findings have important translational implications for preventing treatment failure in ER(+) breast cancer patients currently taking hormonal therapies.

## MATERIALS AND METHODS

### Experimental model

The human breast cancer cell line (MCF7) was obtained commercially from the ATCC. The cell line was maintained in Dulbecco’s Modified Eagle Medium (DMEM; GIBCO) supplemented with 10% HiFBS, 1% Glutamax and 1% Penicillin-Streptomycin, at 37°C in 5% CO2.

### Lentiviral gene transduction

Lentiviral vectors for our gene expression studies were all custom-built to our specifications by GeneCopoeia. The cDNA’s encoding ESR1 (catalogue number: A0322; NM_001122742.1) or ErbB2 (catalogue number: Z2866; NM_004448.2) were inserted into the expression vector Lv-105-puro^R^, containing a puromycin gene resistance cassette. Two vectors encoding ESR1 mutants (Y537S and Y537N) were also generated by site-directed mutagenesis and were confirmed by DNA-sequencing. Packaging cells (293Ta) and all reagents were purchased from GeneCopoeia Inc., respectively. After 48 hours of seeding and culture, 293Ta packaging cells were transfected with lentiviral vectors encoding ESR1, ESR1-Y537S, ESR1-Y537N, ErbB2 or empty vector EV (EX-NEG-Lv105), using Lenti-PacTM HIV Expression Packaging Kit, according to the manufacturer’s instructions. Two days post-transfection, lentivirus-containing culture medium was passed through a 0.45 μm filter and added to the target cells (MCF7 cells) in the presence of 5 μg/ml Polybrene. Infected cells were selected with a concentration of 1.5 μg/ml of puromycin [17]. These cell lines were generated, while working at the University of Manchester, at the Paterson Institute (MF, FS and MPL). Expression of exogenous ESR1 constructs did not detectably alter the expression of endogenous ESR1, as assessed by Western blot analysis.

### Sulfo-rhodamine B (SRB) assay

SRB measures total biomass by staining cellular proteins. After 5 days treatment with of 4-OH-Tamoxifen (4-OHT, Sigma , cells were fixed in 10% trichloroacetic acid (T9159, Sigma) for 1h at 4°C, stained with SRB (S9012, Sigma) for 15 minutes, and washed 3 times with 1% acetic acid (27225, Sigma). The incorporated dye was solubilized with 10 mM Tris-HCl, pH 8.8 (T1503, Sigma). Absorbance was spectrophotometrically measured at 540 nm in a FluoStar Omega plate reader (BMG Labtech). Background measurements were subtracted from all values.

### MCF7 3D-mammosphere formation

A single cell suspension was prepared using enzymatic (1x Trypsin-EDTA, Sigma Aldrich, #T3924), and manual disaggregation (25 gauge needle), to create a single cell suspension. Cells were plated at a density of 500 cells/cm^2^ in mammosphere medium (DMEM-F12 + B27 + 20 ng/ml EGF + PenStrep) under non-adherent conditions, in culture dishes pre-coated with (2-hydroxyethylmethacrylate) (poly-HEMA, Sigma, #P3932), called “mammosphere plates” [36]. Then, the cells were pre-treated for 72 hours with 1µM of 4-OH-Tamoxifen. Vehicle alone (DMSO) control cells were processed in parallel. Afterwards, they were trypsined and seeded in mammosphere plates. Cells were grown for 5 days and maintained in a humidified incubator at 37°C. After 5 days of culture, 3D-spheres >50 μm were counted using an eye piece (“graticule”), and the percentage of cells plated which formed spheres was calculated and is referred to as percent mammosphere formation, and was normalized to one (1 = 100% MSF) [37].

### ALDEFLUOR assay

ALDH activity was assessed in all the MCF7 stable cell lines generated. The ALDEFLUOR kit (StemCell technologies, Durham, NC, USA) was used to isolate the population with high ALDH enzymatic activity by FACS (Fortessa, BD Bioscence). Briefly, after 5 days of culture, the mammospheres were harvested, trypsinized and syringed to generate a single-cell suspension. Then, they were incubated in 1ml of ALDEFLUOR assay buffer containing ALDH substrate (5 μl/ml) for 40 minutes at 37°C. In each experiment, a sample of cells was stained under identical conditions with 30 mM of diethylaminobenzaldehyde (DEAB), a specific ALDH inhibitor, as a negative control. The ALDH-positive population was established, according to the manufacturer’s instructions and was evaluated using 30.000 cells. An ALDEFLUOR-positive signal was detected in Y537S cells, as compared with control (EV).

### Annexin-V analysis

Cell death was quantified by flow cytometry using propidium iodide (PI) and Annexin V-FITC [16]. Briefly, 1,5 x 10^5^ all the transfected cells were plated in 6 multiwell plate in complete media supplemented with 10% HiFBS. Next day, cells were treated with 1µM of 4-OH-Tamoxifen (4-OHT) for 48h and 72h. Vehicle alone (DMSO) for control cells were processed in parallel. After 48 hours, cells were harvested and washed in cold phosphate-buffered saline (PBS). Cells were recentrifuged and supernatants were discarded. Then, cells were re-suspended in 100 µl of annexin-binding buffer. Then, the annexin–FITC conjugate (5 μl) and PI (1 μL) were added and incubated in the dark at room temperature for 15 min. After the incubation period, reaction was stopped by adding 400 μL of annexin-binding buffer. Cells were then analyzed by flow cytometry using a PE Texas Red signal detector for PI staining and a FITC signal detector to detect Annexin V binding. 30,000 events were recorded by FACS using Fortessa BD. Results are the average of three experiments that were performed in triplicate, three times independently.

### Seahorse XFe96 metabolic flux analysis

Real-time oxygen consumption rates (OCR) and extracellular acidification rates (ECAR) rates in all transfected cells treated with 1µM of 4-OHT for 48h were determined using the Seahorse Extracellular Flux (XFe96) analyzer (Seahorse Bioscience, USA) [38]. Briefly, 1,5 x 10^4^ cells per well were seeded into XFe96 well cell culture plates, and incubated overnight to allow cell attachment. Then, cells were treated with 1µM of 4-OHT for 48h. Empty vector (EV) control cells were processed in parallel. After 48 hours of incubation, cells were washed in pre-warmed XF assay media (or for OCR measurement, XF assay media supplemented with 10mM glucose, 1mM Pyruvate, 2mM L-glutamine and adjusted at 7.4 pH). Cells were then maintained in 175 µL/well of XF assay media at 37°C, in a non-CO_2_ incubator for 1 hour. During the incubation time, we loaded 25 µL of 80mM glucose, 9µM oligomycin, and 1M 2-deoxyglucose (for ECAR measurement) or 10µM oligomycin, 9µM FCCP, 10µM rotenone, 10µM antimycin A (for OCR measurement), in XF assay media into the injection ports in the XFe96 sensor cartridge [31,32]. Measurements were normalized by protein content (Bradford assay). Data sets were analyzed using XFe96 software and GraphPad Prism software, using one-way ANOVA and Student’s t-test calculations. All experiments were performed in quintuplicate, three times independently.

### Mitochondrial staining

Mitochondrial activity was assessed with MitoTracker Orange (#M7510, Invitrogen), whose accumulation in mitochondria is dependent upon membrane potential. Mitochondrial mass was determined using MitoTracker Deep-Red (#M22426, Invitrogen), localizing to mitochondria regardless of mitochondrial membrane potential [39,40]. MCF7 transfected cells were seeded for 48 hours. MCF7-EV control cells were processed in parallel. After 48 hours, cells were incubated with pre-warmed MitoTracker staining solution (diluted in PBS/CM to a final concentration of 10 nM) for 30-60 minutes at 37°C. All subsequent steps were performed in the dark. Cells were washed in PBS, harvested, and re-suspended in 300 μL of PBS/CM. Cells were then analyzed by flow cytometry. Data analysis was performed using FlowJo software.

### Label-free unbiased semi-quantitative proteomics analysis

Cell lysates were prepared for trypsin digestion by sequential reduction of disulphide bonds with TCEP and alkylation with MMTS. Then, the peptides were extracted and prepared for LC-MS/MS. All LC-MS/MS analyses were performed on an LTQ Orbitrap XL mass spectrometer (Thermo Scientific, San Jose, CA) coupled to an Ultimate 3000 RSLCnano system (Thermo Scientific, formerly Dionex, The Netherlands) [41]. Xcalibur raw data files acquired on the LTQ-Orbitrap XL were directly imported into Progenesis LCMS software (Waters Corp., Milford, MA, formerly Non-linear dynamics, Newcastle upon Tyne, UK) for peak detection and alignment. Data were analyzed using the Mascot search. Five technical replicates were analyzed for each sample type.

### Ingenuity pathway analysis (IPA)

Unbiased interrogation and analysis of our proteomic data sets was carried out by employing a bioinformatics platform, known as Ingenuity Pathway Analysis (IPA) (Ingenuity systems, http://www.ingenuity.com). IPA assists with data interpretation, via the grouping of differentially expressed genes or proteins into known functions and pathways. Pathways with a z score of > +2 were considered as significantly activated, while pathways with a z score of < -2 were considered as significantly inhibited.

### Quantification and statistical analysis

All analyses were performed with GraphPad Prism 6. Data were presented as mean ± SD (± SEM for OCR and ECAR profiles, see Figures 5 and 6). All experiments were conducted at least three times, with ≥ 3 technical replicates per experiment, unless otherwise stated with representative data shown. Statistically significant differences were determined using the Student’s t test or the analysis of variance (ANOVA) test. For the comparison among multiple groups, one-way ANOVA were used to determine statistical significance. P ≤ 0.05 was considered significant and all statistical tests were two-sided.

### Kaplan-Meier (K-M) analyses

To perform K-M analysis on metabolic gene transcripts, we used an open-access online survival analysis tool to interrogate publically available microarray data from up to 3,455 breast cancer patients. This allowed us to determine their prognostic value. For this purpose, we primarily analyzed data from ER(+) patients that were LN(+) at diagnosis and were of the luminal A sub-type, that were primarily treated with tamoxifen and not other chemotherapy (N = 152 patients). In this group, 100% the patients received some form of hormonal therapy and ~95% of them received tamoxifen. Biased and outlier array data were excluded from the analysis. This allowed us to identify metabolic gene transcripts, with significant prognostic value. Hazard-ratios were calculated, at the best auto-selected cut-off, and p-values were calculated using the log-rank test and plotted in R.

K-M curves were also generated online using the K-M-plotter (as high-resolution TIFF files), using univariate analysis: http://kmplot.com/analysis/index.php?p = service&cancer = breast.

This allowed us to directly perform in silico validation of these metabolic biomarker candidates. The 2017 version of the database was utilized for all these analyses, while virtually identical results were also obtained with the 2014 and 2012 versions.

## Supplementary Material

Supplementary Figures

Supplementary Tables
