# Supplementary material for: The ER-alpha mutation Y537S confers Tamoxifen-resistance via enhanced mitochondrial metabolism, glycolysis and Rho-GDI/PTEN signaling: Implicating TIGAR in somatic resistance to endocrine therapy
Source: Aging (Albany NY). 2018 Dec 20;10(12):4000–23. doi: 10.18632/aging.101690 (PMC6326696; doi:10.18632/aging.101690)
Supplement: Supplementary Figures [file aging-10-101690-s001.pdf]

SUPPLEMENTARY FIGURES

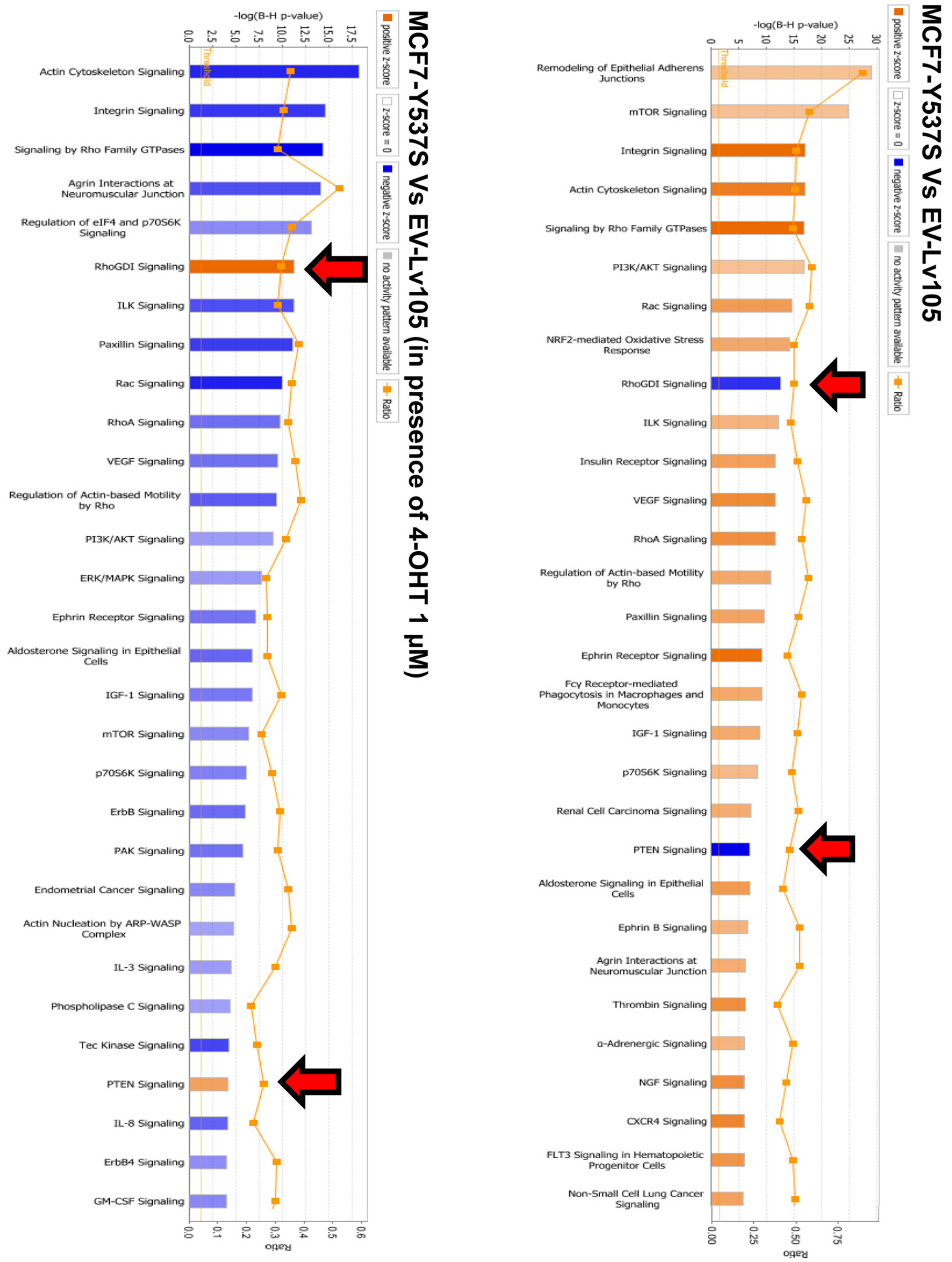

Supplemental Figure S1. **Ingenuity Pathway Analysis (IPA) of proteomics data sets obtained from human breast cancer MCF7-Y537S cells.** (**A**) Canonical pathways predicted to be altered in MCF7-Y537S Vs MCF7-EV-Lv105 are shown. As expected, certain canonical pathways were significantly altered by the differential protein expression in MCF7-Y537S respect MCF7-EV-Lv105. In particular in absence of 4-OHT (1 µM), all the resulting pathways in MCF7-Y537S were up-regulated compared to MCF7-EV-Lv105, except for RhoGDI signaling and PTEN signaling pathways that were down-regulated. (**B**) In presence of 4-OHT (1 µM), all the resulting pathways in MCF7-Y537S were down-regulated compared to MCF7-EV-Lv105, except for RhoGDI signaling and PTEN signaling pathways that were up-regulated. The pvalue ($p < 0.05$) for each pathway is indicated by the bar and is expressed as −1 times the log of the p-value. A positive z-score (Orange color; z-score > 2) represents the upregulation of a specific pathway, while a negative z-score (Blue color; z-score < 2) indicates the down-regulation of a pathway.

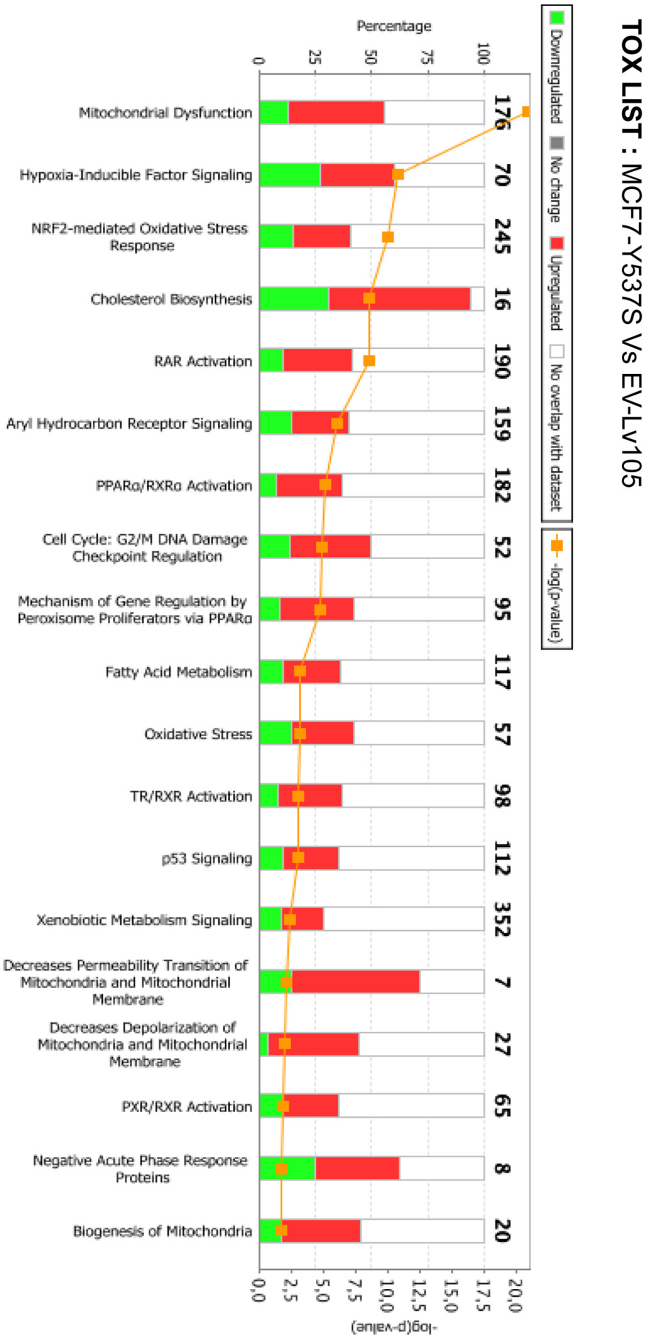

Supplementary Figure 2. Toxicity effects of differentially expressed proteins in MCF7-Y537S Vs MCF7-EV-Lv105. Ingenuity Pathway Analysis showed that certain toxicity functions are significantly enriched by the proteins differentially expressed in this comparative analysis ($p < 0.05$). In the Stacked Bar chart, the p-value for each pathway is indicated by the bar and is expressed as −1 times the log of the p-value (cutoff z-score ± 2).In red color the amount of the proteins up-regulated and in green color the amount of the proteins down-regulated in each patwhays.

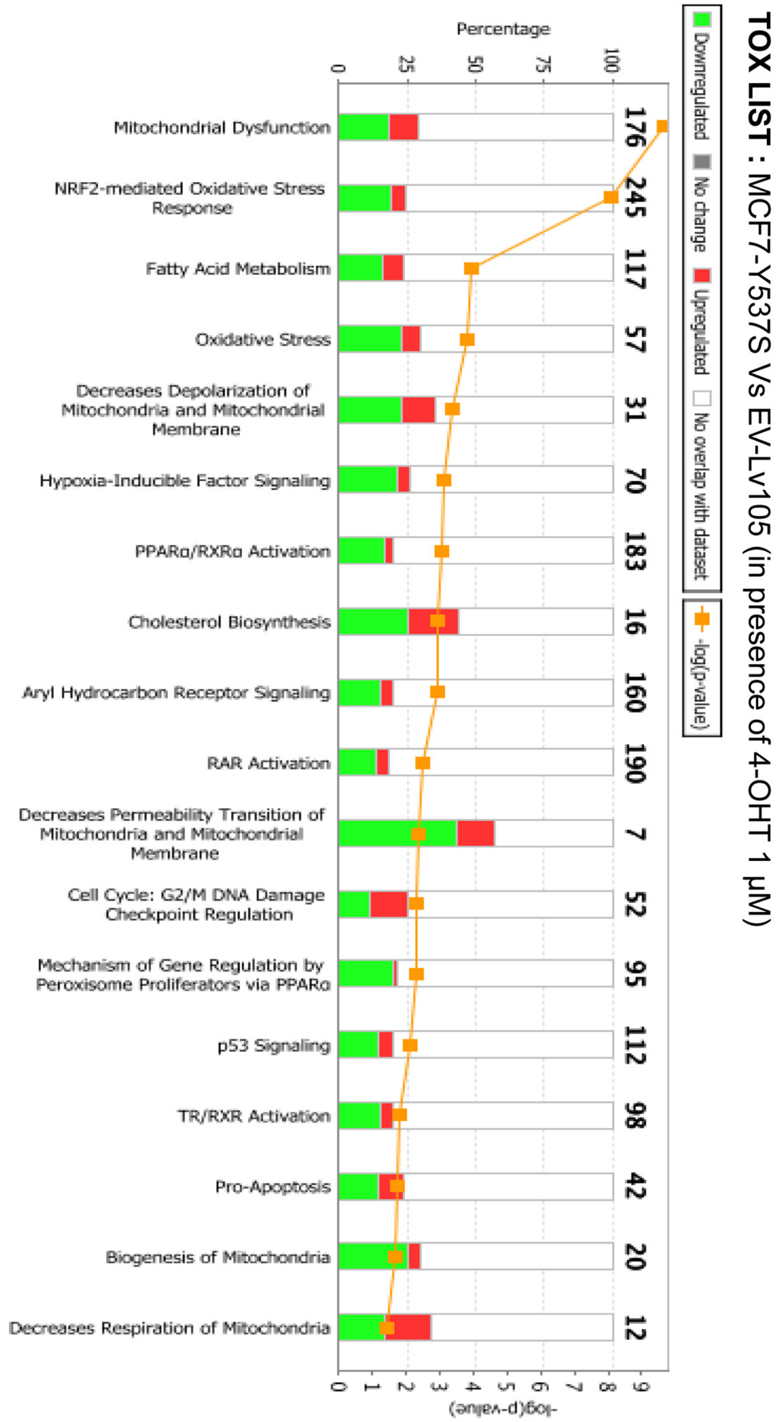

Supplementary Figure 3. Toxicity effects of differentially expressed proteins in MCF7-Y537S Vs MCF7-EV-Lv105 in presence of 4-OHT (1 µM). Ingenuity Pathway Analysis showed that certain toxicity functions are significantly enriched by the proteins differentially expressed in this comparative analysis ($p < 0.05$). In the Stacked Bar chart, the p-value for each pathway is indicated by the bar and is expressed as −1 times the log of the p-value (cutoff z-score ± 2).In red color the amount of the proteins up-regulated and in green color the amount of the proteins down-regulated in each patwhays.

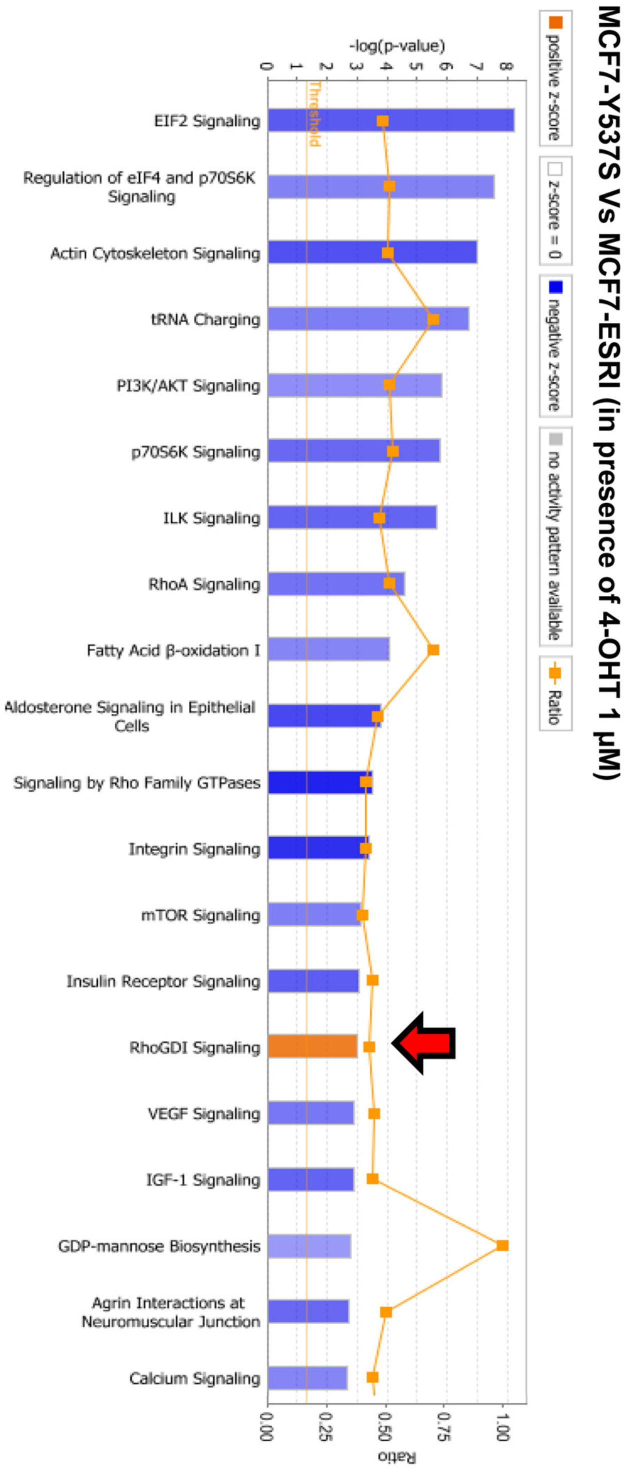

Supplementary Figure 4. Ingenuity Pathway Analysis (IPA) of proteomics data sets obtained from human breast cancer MCF7-Y537S cells compared to MCF7-ESRI. Canonical pathways predicted to be altered in MCF7-Y537S Vs MCF7-ESRI in presence of 4-OHT (1 μM) are shown. As expected, certain canonical pathways were significantly altered by the differential protein expression in MCF7-Y537S respect MCF7-ESRI. In particular, in presence of 4-OHT (1 μM), all the resulting pathways in MCF7-Y537S were down-regulated compared to MCF7-ESRI, except for RhoGDI signaling pathway that was down-regulated.
