# Supplementary material for: The ER-alpha mutation Y537S confers Tamoxifen-resistance via enhanced mitochondrial metabolism, glycolysis and Rho-GDI/PTEN signaling: Implicating TIGAR in somatic resistance to endocrine therapy
Source: Aging (Albany NY). 2018 Dec 20;10(12):4000–23. doi: 10.18632/aging.101690 (PMC6326696; doi:10.18632/aging.101690)
Supplement: Supplementary Tables [file aging-10-101690-s002.pdf]

SUPPLEMENTARY TABLES

Table S1. Mitochondrial-related proteins induced by the ESR1 (Y537S) mutant, as compared with ESR1-WT.

Symbol	Description	Fold-Change (Up-regulation)
**UQCRC2**	Cytochrome b-c1 complex subunit 2, mitochondrial	110.11
HIBADH	3-hydroxyisobutyrate dehydrogenase, mitochondrial	54.89
**NDUFB10**	NADH dehydrogenase [ubiquinone] 1 beta subcomplex subunit	49.59
PGAM5	Serine/threonine-protein phosphatase PGAM5, mitochondrial	8.80
**MRPL47**	39S ribosomal protein L47, mitochondrial	8.27
ACSS1	Acetyl-coenzyme A synthetase 2-like, mitochondrial	7.15
FH	Fumarate hydratase, mitochondrial	6.68
**HSPD1**	60 kDa heat shock protein, mitochondrial	6.02
OGDH	2-oxoglutarate dehydrogenase E1 component, mitochondrial	5.44
**MRPL4**	39S ribosomal protein L4, mitochondrial	4.54
**GRPEL1**	GrpE protein homolog 1, mitochondrial	4.48
ISOC2	Isochorismatase domain-containing protein 2, mitochondrial	3.80
DUT	Deoxyuridine 5'-triphosphate nucleotidohydrolase, mitochondrial	2.93
**SDHB**	Succinate dehydrogenase [ubiquinone] iron-sulfur subunit, mitochondrial	2.75
**NDUFV1**	NADH dehydrogenase [ubiquinone] flavoprotein 1, mitochondrial	2.74
ECSIT	Evolutionarily conserved signaling intermediate in Toll pathway, mitochondrial	2.70
**GATC**	Glutamyl-tRNA(Gln) amidotransferase subunit C, mitochondrial	2.68
**PTRH2**	Peptidyl-tRNA hydrolase 2, mitochondrial	2.35
**DNAJA3**	DnaJ homolog subfamily A member 3, mitochondrial	2.14
**AKAP1**	A-kinase anchor protein 1, mitochondrial	2.07
**HSPA9**	Stress-70 protein, mitochondrial	2.04
**FDXR**	NADPH:adrenodoxin oxidoreductase, mitochondrial	1.99
**TIMM23B**	Putative mitochondrial import inner membrane translocase subunit Tim23B	1.95
**COX4I1**	Cytochrome c oxidase subunit 4 isoform 1, mitochondrial	1.94
**NDUFA5**	NADH dehydrogenase [ubiquinone] 1 alpha subcomplex subunit 5	1.93
**CLUH**	Clustered mitochondria protein homolog	1.88
GLS	Glutaminase kidney isoform, mitochondrial	1.85
**ABCB6**	ATP-binding cassette sub-family B member 6, mitochondrial	1.85
PPA2	Inorganic pyrophosphatase 2, mitochondrial	1.83
**MRPL43**	39S ribosomal protein L43, mitochondrial	1.70
**MRPS16**	28S ribosomal protein S16, mitochondrial	1.69
**MRPL15**	39S ribosomal protein L15, mitochondrial	1.64
**MRPS18B**	28S ribosomal protein S18B, mitochondrial	1.60

Proteins highlighted in **BOLD** are involved in mitochondrial biogenesis.

Table S2. Glycolysis and PPP-related proteins induced by the ESR1 (Y537S) mutant, as compared with ESR1-WT.

Symbol	Description	Fold-Change (Up-regulation)
TIGAR	Fructose-2,6-bisphosphatase (TIGAR)	Infinity
ENO2	Gamma-enolase	128.23
GAPDH	Glyceraldehyde-3-phosphate dehydrogenase	7.41
PKM	Pyruvate kinase	7.41
PHGDHL1	Phosphoglycerate dehydrogenase like 1	4.51
PFKP	Phosphofructokinase, platelet	3.08
ENO1	Enolase	3.07
TALDO1	Transaldolase	2.30
G6PD	Glucose-6-phosphate 1-dehydrogenase	2.19

Table S3. Key signalling molecules induced by the ESR1 (Y537S) mutant, as compared with ESR1-WT.

Symbol	Description	Fold-Change (Up-regulation)
COL6A3	Collagen, type VI, alpha 3	Infinity
ERBB2	Erb-b2 avian erythroblastic leukemia viral oncoprotein 2	14,233.50
STAT3	Signal transducer and activator of transcription 3	28.56
AFP	Alpha-fetoprotein	12.07
TFF1	Trefoil factor 1	3.92
CDK4	Cyclin-dependent kinase 4, isoform	2.82
CD44	CD44 antigen	1.98

Table S4. Y537S targets are transcriptionally up-regulated in breast cancer cells in vivo (Epithelia vs. Tumor Stroma).

Symbol	Gene Description	Up-regulation (Fold-Change)	P-value
**Mitochondrial components**			
FH	Fumarate hydratase, mitochondrial	5.42	7.06E-07
UQCRC2	Cytochrome b-c1 complex subunit 2, mitochondrial	4.84	5.73E-0
SDHB	Succinate dehydrogenase [ubiquinone] iron-sulfur subunit, mitochondrial	4.25	4.24E-05
HSPA9	Stress-70 protein, mitochondrial	3.69	2.64E-04
MRPS18B	28S ribosomal protein S18B, mitochondrial	3.65	2.94E-04
HSPD1	60 kDa heat shock protein, mitochondrial	3.42	5.93E-04
COX4I1	Cytochrome c oxidase subunit 4 isoform 1, mitochondrial	3.39	6.61E-04
AKAP1	A-kinase anchor protein 1, mitochondrial	3.33	7.75E-04
PPA2	Inorganic pyrophosphatase 2, mitochondrial	3.19	1.17E-03
DNAJA3	DnaJ homolog subfamily A member 3, mitochondrial	2.92	2.57E-03
PTRH2	Peptidyl-tRNA hydrolase 2, mitochondrial	2.77	3.82E-03
NDUFA5	NADH dehydrogenase [ubiquinone] 1 alpha subcomplex subunit 5	2.75	4.07E-03
GRPEL1	GrpE protein homolog 1, mitochondrial	2.39	1.01E-02
MRPL15	39S ribosomal protein L15, mitochondrial	2.26	1.39E-02
DUT	Deoxyuridine 5'-triphosphate nucleotidohydrolase, mitochondrial	1.87	3.37E-02
GLS	Glutaminase kidney isoform, mitochondrial	1.81	3.81E-02

Table S4. Continued.

**Glycolytic/PPP enzymes**			
TALDO1	Transaldolase	4.13	6.35E-05
PKM2	Pyruvate kinase 2	3.26	9.79E-04
GAPDH	Glyceraldehyde-3-phosphate dehydrogenase	2.97	2.22E-
ENO1	Enolase	1.96	2.75E-02
**Cell signalling molecules**			
CD44	CD44 antigen	3.44	5.69E-0
CDK4	Cyclin-dependent kinase 4, isoform	2.33	1.19E-02
TFF1	Trefoil factor 1	1.76	4.17E-02

Transcriptional profiling data derived from the analysis of N=28 breast cancer patients are shown, high-lighting the levels of fold-upregulation observed in the epithelial cancer cell compartment (relative to the tumor stroma), and corresponding p-values derived from the analysis of these clinical samples.

Table S5. Mitochondrial-related proteins induced by the ESR1 (Y537S) mutant: Association with Tumor Recurrence.

Symbol	Probe	RFS-HR	Log-Rank Test
HSPD1	200807_s_at	3.40	1.2e-05
HSPD1	200806_s_at	2.36	0.0035
MRPL15	218027_at	3.20	1.7e-05
MRPL4	218105_s_at	2.20	0.005
AKAP1	210626_at	2.19	0.007
AKAP1	201674_s_at	1.90	0.025
PTRH2	218732_at	2.17	0.005
COX4I1	202698_x_at	2.04	0.049
GRPEL1	212434_at	2.01	0.012
HSPA9	200691_s_at	1.97	0.024
MRPS16	218046_s_at	1.96	0.015

RFS, recurrence-free survival.

HR, hazard ratio.

Table S6. Glycolysis and PPP-related proteins induced by the ESR1 (Y537S) mutant: Association with Tumor Recurrence.

Symbol	Probe	RFS-HR	Log-Rank Test
ENO1	201231_s_at	2.28	0.004
TALDO1	201463_s_at	2.14	0.014
TIGAR	219099_at	2.13	0.008
ENO2	201313_at	1.92	0.019

RFS, recurrence-free survival.

HR, hazard ratio.

Table S7. Mitochondrial-proteins induced by the ESR1 (Y537S) mutant: Association with Distant Metastasis.

Symbol	Probe	DMFS-HR	Log-Rank Test
HSPD1	200807_s_at	3.47	9e-05
HSPD1	200806_s_at	2.03	0.03
GRPEL1	212434_at	3.18	0.004
MRPL15	218027_at	2.57	0.0035
MRPS16	218046_s_at	2.53	0.006
COX4I1	202698_x_at	2.26	0.013

DMFS, distant metastasis-free survival.

HR, hazard-ratio.

Table S8. Glycolysis-related proteins induced by the ESR1 (Y537S) mutant: Association with Distant Metastasis.

Symbol	Probe	DMFS-HR	Log-Rank Test
ENO2	201313_at	2.70	0.0035
ENO1	201231_s_at	2.29	0.01

DMFS, distant metastasis-free survival.

HR, hazard-ratio.
